# Philadelphia Hospital—Service of W. W. Gerhard, M. D., Attending Physician

**Published:** 1842-03-19

**Authors:** T. R. Walker

**Affiliations:** Resident


### MEDICAL EXAMINER.

NEW SERIES.

No. 12.] PHILADELPHIA, MARCH 19, 1842. [Vol. I.

### CLINICAL REPORTS.

*Philadelphia Hospital—Service of W. W. Gerhard, M. D., Attending Physician.*

Reported by T. R. Walker, M. D., Resident.

Strictly speaking, all cases of disease are interesting, but in collecting into groups a mass of cases observed in a hospital, we may divide them into two principal classes. First. Those which are admitted under such circumstances as to preclude the possibility of modification by treatment, either from the inherent fatality of the disease, or from the late period, after disorganization, at which the patient is admitted. Second. Cases which illustrate the usual course of curable diseases, and lead us to some therapeutic deductions. In large Hospitals, especially those which, like our Institution, are both Alms-Houses and Hospitals, the number of incurable cases is nenecessarily disproportionately great; for many of the patients enter only after having exhausted their money, and their hope of recovery: in private practice, on the other hand, the proportion of fatal cases is trifling. Although it is not agreeable in itself to report cases terminating unfavorably, yet they are, perhaps, of more real instruction to the practitioner than those which end in recovery, and throw as much, if not more, light upon our knowledge of disease, and indirectly of its treatment.

The present report includes the number of patients under cure in four wards of the Philadelphia Hospital; three of them for men and one for women.

The number treated between the 10th of January and the 1st of March, 1842, is 139. Of these, 84 were white males; 38 black males; and 17 white females. During this time, 71 were discharged; 18 died, one half of whom consisted of cases of pulmonary phthisis. The remainder are still in the wards.

| | | |
|---|---|---|
| Thoracic Diseases. | Phthisis Pulmonalis, - - - | No. 19 |
| | Pneumonia, - - - | " 5 |
| | Pleuro Pneumonia, - - - | " 2 |
| | Bronchitis, - - - | " 11 |
| | Pleuritis, - - - | " 5 |
| | Disease of Heart and Aorta, - - | " 7 |

Of the cases of phthisis included in the foregoing table, all were far advanced at the time they entered. Nearly all offered the signs of cavities already formed in one or both lungs; nine have been carried off by the disease. One case only offered many points of interest.

Case I. A weaver, æt. 27, of intemperate habits, admitted January 21, 1842. Has been very healthy till last August, when, after great exertion at a fire, was overheated and fatigued, lay down upon the grass. Chilliness soon followed, and the next morning he felt a lightness of the head. He also suffered from considerable cough. This disappeared in a few days, but soon returned. Has had no pain in the chest except for the last three months, when, upon rising from bed, there was uneasiness at the left nipple; none scarcely at the right. In August began to expectorate creamy phlegm. For the last two months his expectoration has been nummular. Was obliged to quit work in November on account of increased pain, cough, emaciation, and failure of strength.

January 22. As first seen, emaciation and debility great. Appetite bad for last three weeks. No fever or chill, and complains of no pain. Bowels opened naturally. Pulse small and frequent. Respiration about thirty-six in the minute, and somewhat laboured.

*Physical Signs.*—Respiration, posteriorly at summit of left lung is harsh. At summit of right lung some crackling, almost cavernous. Respiration below this very feeble, scarcely audible. Percussion over the same extent decidedly flat, with slight dilatation of the chest. Anteriorly beneath the left clavicle was distinctly heard amphoric respiration. Blister to right side.

As the case was evidently one of very advanced phthisis, with large pleuritic effusion upon the right lung, a palliative treatment was adopted. The patient was placed upon the infusion of digitalis, and a blister was applied to the right side of the chest. The digitalis producing no decided effect, it was discontinued after some days, and the patient placed upon a simple infusion of the wild cherry bark, acidulated. A partial absorption of the effused fluid took place, but with very little relief to the patient. The symptoms continued until the 12th of February.

Feb. 12th. No important change has been observed, except that he suffered for one or two days from slight diarrhœa. His pulse all the time ranged from 130 to 160. Respiration was high and impeded. Appetite bad. Now his cough is much worse. Expectoration (of thick mucopurulent matter) more abundant. Slight delirium, of which the patient is himself conscious. More pale, slight tremors. Pupils rather contracted. Complains of giddiness in the head. Profuse sweat and intense fever.

February 14. Has no delirium, but is very dull. Pulse is 136 and feeble. Respiration high and difficult. Tongue not much affected, slightly red and moist. Bowels are quite loose. There is pain in the chest. Gurgling heard at upper part of right lung. Sonorous and subcrepitant rhoncus heard in the left lung, particularly towards its lower portion. This is probably the consequence of commencing softening.

15. Delirium of an active character having recurred on the evening of the 14th, a blister was applied to the back of the neck. It produced but temporary relief. From the first appearance of this symptom he sank rapidly. Countenance grew more pale and anxious. Difficulty of breathing increased. Subsultus and tremors were more evident. His symptoms were of this oppressive character until the evening of the 15th, at which time he expired.

No autopsy could be made, but the case was evident enough as regarded the pectoral symptoms. The only question of interest was the cause of the delirium. This symptom is a very frequent one towards the latter stages of phthisis. In some cases it depends upon the mere nervous prostration which may end in delirium; but in others it is a true arachnitis, and the patient dies of the effusion of lymph, and of the tuberculous deposit in the membranes of the brain, especially at the base. The serous inflammation of the brain is most apt to occur when the pleura or some other serous membrane is inflamed; an example of the repeating inflammations, if we may so call them, of serous membranes.

Of the cases of pneumonia, all terminated favourably. The following case was severe from the rapid development of cerebral symptoms.

CASE II. *Pneumonia of Left Lung. Severe Symptoms. Epistaxis notwithstanding bleeding. Cerebral Symptoms. Treatment. Venesection. Cupping repeated. Infusion of ipecac. and blue mass. Subsidence of symptoms on 13th day coinciding with ptyalism.*

William Pool, æt. 23, entered ward on the 7th February, 1842, from the venereal ward, where he had been for five months past with syphilis. Was taken ill January 31 with a sudden and severe cough; hoarseness, but no pain. Suffered from chills on the 2d, 3d, and 4th of February, with some pain. Took to his bed on the 2d of February. Has been in and out of it till his entrance into medical ward. No treatment.

State on the 7th. Of muscular, full frame. Light hair. Face greatly flushed. Skin very warm and dry. Some dilatation of nostrils. Respiration frequent, about 32. Pulse 120, full and resisting. Pain is felt at right side, chiefly under the sternum. Expectoration frothy, transparent, and moderately viscid. Tongue red at point, coated at edges, and rather dry. Abdomen soft.

*Physical Signs.*—Posteriorly on left side, respiration for the lower two-fifths is rude. Intensely tubal at middle. Slight crepitus heard in the middle also. Anteriorly the impulse of the heart is strong. First sound slightly roughened.

℞ Venesection.
Infus. Ipecac.

8th. Has not slept. From some mistake as to the quantity of the infusion directed at a dose, has vomited several times. Epistaxis has been quite free for several hours. Flush of face is yet present. More diffused than usual. Skin warm, but not very dry. Bowels not open for 24 hours. Pulse 116, and of good impulse.

9th. Has passed a more comfortable night. Still some pain at lower part of left side. Tongue more moist. Skin of nearly natural temperature. Pulse 118, and full. Respiration not counted. Vomiting relieved by the application of sinapisms, and the epistaxis arrested by the tincture of creosote applied to the nostrils.

℞ Cut cups over seat of pain, No. vj.
Infusion continued.

10th. Countenance expressive of great distress. General redness of the face. Has passed a disturbed night. Delirious a greater portion of it. Slept a short time after cups were applied. His skin this morning is warm. Complains of nausea, though the infusion has been stopped since early last evening. Some tenderness over the epigastrium. Thirst is considerable. Tongue not much furred, and is moist. Throat very dry. Bowels not open since the injection which was administered the evening of the 8th. Respiration is very difficult, irregular, and upwards of thirty. Pulse quick, 128. Expectoration is slight and frothy, not of much tenacity. Percussion posteriorly of left side is very dull till near the summit. Subcrepitous rhoncus heard over lower lobe. Bronchial respiration at summit of left lung.

℞ Common enema and blue pill, gr. i., q. s. h.

Evening, 8 o'clock. Not at all improved. Pulse 130. Respiration difficult, and 50. Is delirious, indisposed to talk, but puts his hand upon his abdomen when questioned as to the seat of pain. Vomiting has ceased and bowels been opened since morning. Cough is oppressive and expectoration very difficult.

11th. Face flushed without any positive blush of cheek. Frequent retching. Cough. Expectoration very scanty, slightly tenacious and sticks in his throat. Respiration 49, and variable. Pulse 120, and soft. Tongue red, rather dry at tip, coated at centre. Nausea and loss of appetite. Great thirst.

Respiration in upper portion of left lung is rude and feeble. Strongly bronchial from the top of the lower lobe. At the middle of lung intensely tubal. Abundant crepitous rhoncus heard in the axilla. Treatment continued.

12th. Is more comfortable. Still feels pain in left side. Has slept quite well. Vomits after eating. Cough and expectoration troublesome. Skin cool and moist. Tongue moist and a little coated. Respiration still high, 48. Pulse small, 110. Bowels not open since injection of yesterday. Ptyalism very slight.

℞ Cut cups, No. vj. over left side ;
No. iv. over epigastrium, and a common enema.

13th. Has slept well all night. Ptyalism is very evident. Cough much less troublesome. No improvement in the power of expectorating. Skin is cool and moist. Pulse little over 80. Respiration more easy.

℞ All medicines stopped.

14th. Since the appearance of ptyalism all the symptoms are ameliorated. Expression more natural. Skin cooler. Pulse full and soft, 84. Respiration about 24. Expectoration much more abundant.

18th. From the 14th to this time the improvement of the patient has been continued. His pulse has gradually fallen to its healthy state. He has enjoyed natural sleep. Appetite has improved. Tongue become perfectly clean. This morning we find him a little more indisposed; the consequence of the nurse's allowing him to leave his bed. A blister is applied over the left side, and the decoction of senega ordered.

23d. After he recovered from the exhaustion of rising, his convalescence continued.

25th. Discharged well.

This is an instance of pneumonia with strong determination to the head, which a free bleeding was not sufficient to arrest, but a natural flow of blood, which in all was not less than a pint, removed the symptoms. A return of them took place, for which cups were applied. The entire recovery of the patient took place as soon as ptyalism was produced. Was this not a direct result of the mercury? Or is the successful termination of the case to be attributed to other causes? The danger of the patient appeared to depend not upon the pectoral symptoms, but on the cerebral determination. Still we have every reason to believe that the mercurial action hastened the cure, acting as it usually does in pneumonia after the acute conjestion has been relieved by free abstractions of blood.

By pleuro-pneumonia is meant cases of pneumonia in which the pleuritic effusion is disproportionately large. Of the two cases, one, that of a black, who entered in a state of great exhaustion with pneumonia of the left lung, and very large effusion into the right pleura, terminated fatally. The period for treatment had passed previously to the entrance of the patient. The other case is one of some interest.

Case III. *Pleuropneumonia.*—S—— C——, æt. 30, admitted February 5th, 1842. Was taken ill on the morning of the 5th. About 4 o'clock he went to his work, but soon felt too unwell to continue working. For the previous week had felt an uneasiness a little to the right of lower part of the sternum. Had, during this time, a bad cold. His appetite did not leave him. His cough was slight for several weeks before this. On the morning of the 5th a sharp and severe pain seized his head and neck. He also suffered from chills, and pain in the breast.

State on the evening of the 5th. His face is much flushed,—a diffused redness. Pain of right side very acute. Dyspnœa considerable. Bowels constipated. Pulse much excited, but not strong. Skin warm and dry. Cough short and hacking. Some cephalalgia. No expectoration. Appetite gone. Slight jaundice. Decubitus dorsal, and slightly inclined to the right side.

℞ Cut cups, No. viij. to right side,
and a Dover's powder ordered to be taken.

The next morning an infusion of eupatorium and serega were directed for him.

7th. Slight jaundice discovered about the eyes. Decubitus still dorsal and inclined to the right side. Some dilatation of nostrils. Moderate flush of cheeks, especially of right. Sleeps badly. Intelligence clear. Cough short, dry, and interrupted. Tongue white at centre. Bowels open freely after the oil which was taken this morning. No epigastric soreness. Skin is moist and of moderate temperature. Pulse is 100, rather feeble, and slightly irregular. Percussion on right side posteriorly dull throughout. Respiration of the same is feeble at middle portion, slightly bronchial at summit. No well defined crepitus heard. Action of the heart is feeble. The respiration anteriorly of right side is rude and puerile, with slight traces of crepitus. Percussion dull throughout.

℞ Infus. eupatorii et ipecac.
Cut cups, No. vi. to right side.

8th. Pulse is 72. Respiration 22. General appearance much improved. Flush of face is diminished. Skin cool and moist. Slept well.

Respiration posteriorly feeble at lower portion of both lungs, almost inaudible at lower third of right lung. Slight friction sound heard. Percussion as when previously examined, though it may be a little less dull. Treatment continued.

9th. Flush of face still present. Countenance still disturbed. Eyes jaundiced. Tongue moist and coated with a white fur. Complains very much of increased pain in the chest. Cough is dry and paroxysmal. Dyspnœa, with dilatation of nostrils. Sighs frequently and breathes very irregularly. Bowels are free. No expectoration. Percussion anteriorly on right side is dull, becoming flat as we approach the lower third. Posteriorly it is flat and the respiration is very feeble. No crepitus heard. Pulse is 100.

℞ Cut cups, No. vj. to side ;
Infus. continued.

10th. At the evening visit of yesterday his symptoms were much aggravated. Pain of right side was very acute, with corresponding dyspnœa. Cough frequent, dry, and short. Cups were again applied to the side, after which immediate relief followed. Now the skin is cool and moist. He is disposed to sleep. Tongue is moist. Pulse is of good strength, 80. Respiration 30. Treatment continued.

11th. Is able to sit up. Jaundice very slight. Cough much less. No pain in the side. No expectoration. Respiration easy and natural. His improvement progressed rapidly without further medicative treatment, and by the 15th he was well enough to be discharged.

In this case we had irritation of the liver coinciding, as it often does, with pleurisy of the right side. The patient's countenance was jaundiced, and the general expression was that of dyspnœa, with inability to lie on the right side. As the pleurisy was not attended with much effusion, and the febrile excitement was slight, cupping answered a better purpose than general bleeding would have done. The infusion of ipecacuanha is one of the most frequeut hospital prescriptions in slight pectoral affections.

